# The Food of the Ancients, and of Modern Infants

**Published:** 1877-09

**Authors:** William A. Green

**Affiliations:** Macon, Ga.


### SELECTIONS.

### THE FOOD OF THE ANCIENTS, AND OF MODERN INFANTS.

BY WILLIAM A. GREEN, M. D., MACON, GA.,

After writing several papers on modern pharmaceutical and chemical science—detailing many new remedies and compounds—it is really pleasant to turn our mind and thoughts from nauseous physic to the more palatable subject of diet—something to feed the physical system. The subject of food for mankind is a peculiarily interesting one— whether viewed in the light of medicine or otherwise. The ancients, during the period of their highest civilization and prosperity, devoted far more attention to this subject than we moderns, and yet, strange to say, left on record less evidence of scientific research concerning the food they ate than any other matter intimately connected with their history. Man, destined by Providence to wander over the globe, and to live in various climes, is essentially an omnivorous animal. According to the country he inhabits, its productions, and the nature of his pursuits, his mode of living differs. The inhabitant of cold and sterile regions, on the borders of the ocean, becomes *ichthyophagous* ; and fish—fresh, fried, smoked or salted —is his principal nourishment. The bold huntsman lives upon the game he pursues; while the nomadian shepherd, who tends his herd over boundless steppes, supports himself on the milk of his flock. In warm countries, fruits and vegetables constitute the chief snpport of life; and there the disciples of Pythagoras can luxuriate in the rich product of a bountiful soil, solely debasing themselves from *beans*, which, like all flesh, they consider to have been created by putrifaction. What would these good disciples have done among the Scythians and the Gauls, who, according to Sidonius Apollinaris, mingled blood and milk for food? *Solitos que cruentum. Lac potare Gitas, ac pocula tingere venis*—or the stunted natives of the Artic regions, who feed upon whales and seals, drink deep potations of train oil, and consider the warm blood of the seal an exquisite beverage, dried herrings, moistened with blubber, a dainty, and the flesh of the seal, half frozen in snow during the winter, or half corrupted in the earth in summer the most delicious morsel. In some desolate tracts of the globe where food is very scarce, we find the wandering Indian satisfying his cravings with earth and clay; and Humboldt informs us that the Ottomaques, on the banks of the Mata and the Oronoco, feed on a fat unctious earth, in the choice of which they display great epicurean skill, and which they kneed into balls of four or six inches in diameter, and bake slowly over the fire. When about to be used, these clods are soaked in water, and each individual consumes about a pound of them in a day. It is in temperate regions that man displays his omnivorous propensities; there animal food can be abundantly procured, and every description of grain, roots and fruits easily cultivated.

Various philosophers, in idle disquisitions, have endeavored, by the most absurb hypotheses, to determine what is the natural food of man, and to show that he is not created *omnivorous*. The comparison between our species and animals confutes these vain theories. Man, by his natural structure, was created omnivorous, and there is no doubt but that a judicious, mixed alimentation is the best calculated to insure health and vigor, and enable the ambitious or the industrious wanderer to spend his winters near the poles, colonize beneath the equator, or inhabit regions where the hardiest of animals must starve and die. The masticative and digestive organization of man assigns to him an intermediate rank between carnivorous and herbivorous creatures. It is to the abuse of this omnivorous faculty, that Providence has bestowed upon mankind, that we owe many of the diseases under which our species labors. "*Multos morbos, multa fercula fecerent*," sayeth Seneca; yet we are far more temperate in the present age than the ancients during the period of their high civilization and prosperity. Their exesses must have been of the most disgusting nature, since, to indulge more easily in their gluttonous propensities, they had recourse to *emetics* both before and after their meals—"*vomunt ut edant, edunt u' vomant, et equlas quas tolo orbe conquirunt nec concoquere degnantur*," was the reproach of the above quoted philosopher. Sentonius and Deon Cassius give Vitellius the credit of having introduced this revolting custom into fashion; and splendid vessels of gold and silver for the purpose were introduced in their feasts. Then, the sums expended by the ancients on their tables exceed all belief. Vitellius, the prince of gluttons, expended upwards of £3,200 daily, and some of his repasts cost £40,000. At some of them, according to Seutonius, 700 birds and 2,000 fish were served up. Actius Verus laid out 600,000 *Sestertii* on one meal; and some of the dishes of Heliogabalus cost about £4,000 of money. The freaks related of this emperor are scarcely creditable; but his gastronomic profession may be easily conceived when we find that his very mats were made with the down of hares, or the soft feathers of partridges. Philoxemus wished he had the throat of a crane, that he might prolong the delights of eating.

The diversity of *substances* which we find in the catalogue of articles of food among these ancients, is as great as the variety with which the art or the science of cookery prepared them. The cooks of the ancients appear to have been much more consummated in their art than our modern practitioners. Athæneus records various descriptions of their incomparable science. A new dish immortalized its inventor, and transmitted its name to prosterity. Philosophers and poets gloried in their culinary science; the pleasures of the table were the subject of their writings, their song and their verse.

So refined was the *taste* of the ancient *bons vivans*, that Montanus, according to Juvenal, would proclaim, at the *first bite*, whether an oyster was an English product or not. These ingenious gluttons had recourse to every experiment that could add to their enjoyment. Philoxemus, and many others, used to accustom themselves to swallowing *hot water*, that they might be able to attack scalding dishes before less fire proof guests would dare to taste them. Sinon maintained that cookery was the basis of all arts and sciences; natural philosophy taught us the seasoning of dishes; architecture directed the construction of stoves and chimneys; the fine arts, the beautiful symmetry of each dish. He applied the principles of war to the drilling and marshalling of cooks, confectioners and scullions—posting proper sentinels to watch the fire and videttes to keep off idle intruders. He said that man is a "cooking animal"—and a proper bill of fare he considered the *ne plus ultra* of human genius. Their tastes and selections of substances for food was peculiar and various. Mæcenas delighted in donkey-flesh, and compared the wild ass to venison. Plutarch tells us the gravid sow was considered a delicious mess, fit for the Gods. Catius tells us how to drown fowls in Falernian wine to render them more luscious and tender. Several fishes were immortalized as articles of food. The muræna helena was educated in their ponds, and rendered so tame, that he came to be killed at the tinkling of his master's bell or the sound of his voice. Hertius, it is said, ceeded six thousand of these fish to Cæsar as a great kindness and favor.

*Snails* were a great dainty. Fulvius Herpinus was immortalized for the discovery of how to feed them on bran and other articles, and Horace informs us they were served up, broiled on silver gridirons. Grasshoppers, locusts and various insects, were equally acceptable to our first gastronomic legislators. Swans and cranes were first deprived of their sight, by the Romans, in order more effectually to *fatten* them. The modern gastronomist perhaps is not aware that it is to the ancients he owes his dilicious fattened duck and goose livers—the inestimable *foies gras* of France. But the Romans certainly knew not the *turkey*, for they are no where mentioned in all their history (a fact not generally known) —this gift we moderns owe, I think, to the *Jesuits*.

The singular influence which *climate* exercises over the quality and properties of food, especially *vegetable*, is interesting and instructive. We have not space to dwell on this part of our subject. But we find plants that are *poisonous* in some countries, edible and wholesome in others.

*Culture* and soil modify plants in a singular way. Flowers which yield a powerful perfume in some latitudes, are inodorous in others, and according to climate, their odor is distressing or pleasant. *Cultivation* brings forth singular intermediate productions. By its magic power we have seen the coriaceous and bitter almond transformed into the luscious peach, the sloe into the delicious plum, and the common *crab* into the golden *pippin*. The celery sprung from the nauseous and bitter *apium graveoleus*, and the colewort is metamorphosed into the cabbage and the cauliflower. All cruciform plants degenerate within the tropics, but acquire increased energies in cold countries.

No where in ancient history do we find the sentence devoted to food for innocent infantile life—all for mankind—and we see to what ridiculous and disgusting extremes these licentious gastronomers carried their excesses. Diffusion of knowledge and habits of industry have overcome all these gluttonous propensities —and all the sciences, especially chemistry, have done much to improve our food, its mode of production and manner of preparation. In these modern times our pharmacists have not neglected and forgotten the infant, the little children, as did our ancient brethern. Our Savior, when on earth, remembered them when He said: "Let little children come unto me."

Many have been the efforts to manufacture a suitable diet for the little creatures, a few approximating the mother's milk— something to suit their delicately organized stomachs, and that they can digest and thrive on. It has been greatly needed especially in these latter days of fashion, of pride, and of high life, when mothers refuse to nourish their own offspring, or trust them to "*wet nurses*," or the artificial food sold by druggists. But it frequently happens that infants must be reared with this food, because of death or disease of the mother, when it is of the utmost importance that we be able to select the *best* the market affords—and I desire to give my own experience in this particular. In a practice of 25 years, I have used all the preparations I have heard of—and for the past few years have been prescribing what is known in the drug stores by the name of *papoma*, a nutritious and easily digested food for infants as well as an excellent diet for adult *invalids*. The foundation for good health, a happy life and ripe old age, or for suffering and premature decay, is always laid in infancy and childhood. Numerous articles have been forced to extensive sale pretending to answer these requirements—but none are superior and few equal to this preparation, which is manufactured by John Wyeth & Bro., of Philadelphia, to whom we are indebted for so many valuable and reliable pharmaceutical preparations. This firm, and their articles, are highly recommended to the profession by the American Pharmaceutical Association,—and the process of the manufacture of papoma in the different stages of preparation was exhibited by the late Prof. Edward Parrish to the members of the American Medical Association when it convened in Philadelphia, in 1872, and attracted very favorable attention, and was highly commended. This commendation in the best testimony that could be given, the Association being composed of the most eminent medical men in the United States. Children to whom I have administered papoma always have an easy digestion and an available supply of what their tissues and organs require for nourishment and growth. We all know that meat extracts and juices are very unsuitable for very young children and do not meet the requirements of the infant, upon whom they act as stimuli only. Papoma is strictly vegetable, and I ask in confidence the most thorough trial of it, assured it will make good all I have written concerning it. It is true we do not often need such a preparation, but when required it is sudden, and frequently unexpected—therefore the greater necessity for having in our minds the best and most reliable article of the kind. I often wonder how our ancient brethern in medicine, in the day of high civilization and great refinement, got along without some *food* for *infants*, as I have never seen any mention of anything of the kind in reading the medical history of those times—yet, as we have seen, they spared neither time or money in furnishing themselves, even to the most ridculous extreme.—*Cincinnati Lancet and Observer.*

